# Plasminogen regulates mesenchymal stem cell–mediated tissue repair after ischemia through Cyr61 activation

**DOI:** 10.1172/jci.insight.131376

**Published:** 2020-08-06

**Authors:** Hao Duan, Zhenqiang He, Maohuan Lin, Yanling Wang, Fan Yang, R. Alan Mitteer, Hyun-Jun Kim, Eujing Yeo, Hongyu Han, Ling Qin, Yi Fan, Yanqing Gong

**Affiliations:** 1 Division of Translational Medicine and Human Genetics, Department of Medicine, and; 2 Department of Radiation Oncology, Perelman School of Medicine, University of Pennsylvania, Philadelphia, Pennsylvania, USA.; 3 Department of Neurosurgery, Sun Yat-sen University Cancer Center, State Key Laboratory of Oncology in South China, Collaborative Innovation Center for Cancer Medicine, Guangzhou, China.; 4 Department of Cardiology, Sun Yat-sen Memorial Hospital, Sun Yat-sen University, Guangzhou, China.; 5 Department of Orthopaedic Surgery, Perelman School of Medicine, University of Pennsylvania, Philadelphia, Pennsylvania, USA.

**Keywords:** Vascular Biology, Plasmin, Stem cell transplantation

## Abstract

Stem cell transplantation has emerged as a promising strategy in regenerative medicine. However, the poor survival and persistence of the transplanted cells, including mesenchymal stem cells (MSCs), in the hostile ischemic microenvironments represents a major therapeutic barrier. Here we report that plasminogen (Plg) stimulated MSC functions and promoted MSC survival during tissue repair after ischemia. Genetic Plg ablation abolished MSC survival, migration, and proliferation in mouse ischemic limbs, and abrogated MSC-mediated blood reperfusion, neovascularization, and tissue repair after ischemia, suggesting a critical role for Plg in MSC-mediated tissue repair. Furthermore, multiplex cytokine array analysis identified that Plg cleaved and activated cysteine-rich protein 61 (Cyr61), an ECM-associated growth factor, to stimulate MSC survival and migration. Overexpression with truncated Cyr61 in MSCs rescued blood reperfusion after hind limb ischemia in Plg-deficient mice. Finally, Plg-mediated Cyr61 cleavage promoted endothelial cell migration and neovascularization in vitro and in vivo. Our study reveals that Plg promotes MSC survival, persistence, and paracrine effects and improves postischemic neovascularization and tissue repair through Cyr61 cleavage and activation. Thus, targeting Plg/Cyr61 may offer exciting therapeutic opportunities for strengthening MSC therapy in ischemic diseases.

## Abstract 2

Plasminogen promotes mesenchymal stem cell function and improves post-ischemic neovascularization and tissue repair through cysteine-rich protein 61 activation.

## Introduction

Mesenchymal stem cells (MSCs) are multipotent stem cells that possess the potential to self-renew and differentiate into a variety of specialized cell types, such as osteocytes, adipocytes, chondrocytes, fibroblasts, and endothelial cells (ECs) (1–4). In addition to their ability of pleiotropic differentiation, MSCs exhibit a set of fairly unique properties, including antiapoptosis, proangiogenesis, production of growth factors, neuroprotection, antifibrosis, and chemoattraction (5). Therefore, these pleiotropic properties of MSCs provide a broad spectrum for their potential in regenerative therapies.

The approach that uses MSC-mediated tissue repair has been one of the most promising treatments for ischemic diseases (6, 7). As an alternative to traditional treatment strategies in patients with ischemic diseases, cell therapy that aims at attenuating fibrosis, promoting tissue repair, and inducing neovascularization, thereby likely restoring the supply of blood to the ischemic tissue to form a tissue repair–friendly microenvironment, has been drastically exploited in recent years (7, 8). Preclinical and clinical trials have shown the great potential of MSC therapy, including variable benefits to mice or patients with ischemic heart disease as well as peripheral artery disease (9–12). However, clinical trial failures have been frequently reported, largely due to low retention and survival of the transplanted MSCs in the ischemic environment, compromising long-term efficacy of MSC therapy (13–15).

Plasminogen (Plg) is a major enzymatic component of the fibrinolytic system that is converted to the active enzyme plasmin by tissue plasminogen activator (tPA) or urokinase plasminogen activator to degrade fibrin clots formed within blood vessels (16). As a thrombolytic agent, tPA has been used as the first line of treatment of acute myocardial infarction (MI) for almost 2 decades. In addition to its canonical thrombolytic function, we previously showed that Plg is crucial for hematopoietic stem cell–mediated cardiac repair and neovascularization after MI (17). However, the role of Plg in MSC-mediated tissue repair remains largely unknown.

Cysteine-rich protein 61 (Cyr61)/CCN1 is an ECM-associated signaling protein of the CCN family. During embryonic development, Cyr61 is critical for cardiac septal morphogenesis, blood vessel formation in placenta, and vascular integrity (18). In adulthood, Cyr61 plays an important role in inflammation and tissue repair (19–21). Interestingly, Cyr61 is abundantly present in the culture medium of MSCs and is required for MSC-mediated angiogenic responses (22), suggesting a potential role for Cyr61 in MSC-mediated tissue repair. However, how Cyr61 activity is regulated in MSCs and MSC-mediated tissue repair after ischemia remains unclear.

In this study, using stem cell tracking approaches in an experimental mouse hind limb ischemia (HI) model with genetic Plg ablation, we reveal that Plg is critical for MSC survival and persistence in ischemic tissues and regulates MSC-mediated neovascularization and tissue repair. Moreover, our data uncover that truncated Cyr61 cleaved by Plg in MSCs substantially enhances MSC viability and migration and MSC-mediated neovascularization. These findings suggest Plg as a vital therapeutic target for MSC-based cell therapy in ischemic diseases.

## Results

### Plg promotes MSC proliferation, survival, and migration.

**Figure 1:**
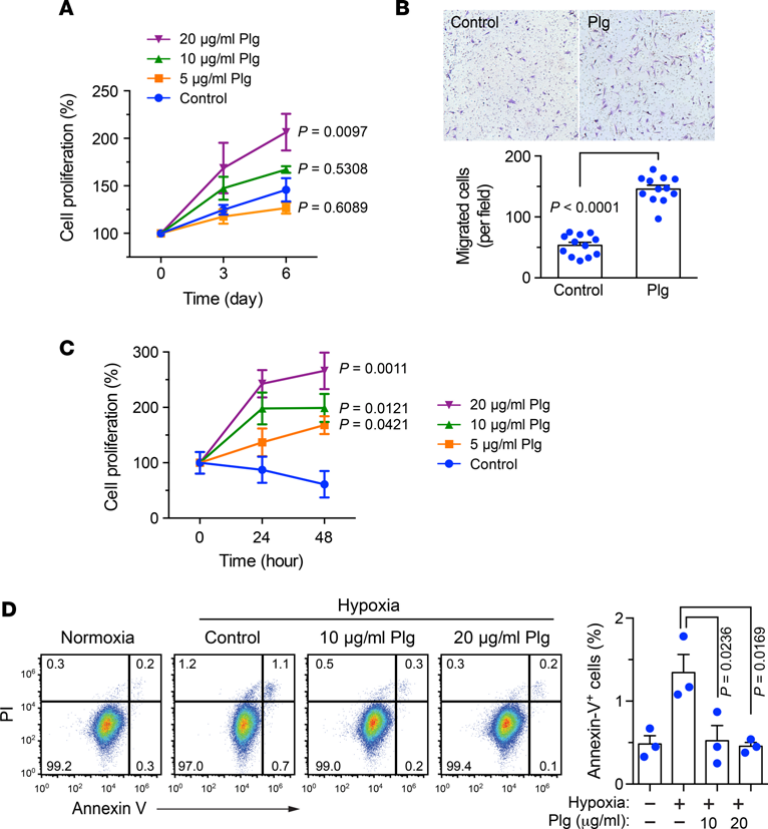
Plg promotes MSC proliferation and migration under normoxia and improves MSC survival under hypoxia. (**A**) MSCs were cultured with serum-free medium and treated with or without Plg under normoxia. Cell proliferation was determined by MTS-based assay (*n* = 4–8, mean ± SEM). Statistical analysis by 1-way ANOVA (compared with control). (**B**) MSCs were suspended in serum-free medium supplemented with or without Plg and seeded on Transwell membranes precoated with Matrigel. Plg-depleted FBS was used as the attractant to induce the migration through Matrigel. Cell migration was determined after 4-hour incubation. Top, representative images. Bottom, quantified results (*n* = 12, mean ± SEM). Statistical analysis by unpaired Student’s *t* test. (**C**) MSCs were cultured with serum-free medium and treated with or without Plg under hypoxia (2% O_2_). Cell proliferation was determined by MTS-based assay (*n* = 3, mean ± SEM). Statistical analysis by 1-way ANOVA (compared with control). (**D**) MSCs were cultured with serum-free medium and treated with or without Plg under hypoxia (2% O_2_) for 24 hours. Cell apoptosis was analyzed by using FITC-annexin V–based flow cytometry. Left, representative sorting. Right, quantified results (*n* = 3, mean ± SEM). Statistical analysis by 1-way ANOVA. Experiments were repeated 3 times and representative results are shown.

The survival and engraftment of transplanted MSCs in ischemic environments are crucial for their therapeutic efficacy in treating ischemic diseases (6). We initially explored the role of Plg in MSC functions. MSCs were isolated from mouse bone marrow and characterized as Sca-1^+^CD90^+^CD11b^−^CD117^−^ adherent cells with a spindle-shaped morphology (Supplemental Figure 1; supplemental material available online with this article; https://doi.org/10.1172/jci.insight.131376DS1). Our data showed that Plg stimulated cell proliferation in mouse MSCs in a dose-dependent manner (Figure 1A). Moreover, Plg markedly enhanced MSC migration in response to FBS (Figure 1B), suggesting a role for Plg in stimulus-dependent regulation of MSC motility and viability under normoxia. To test whether Plg protects MSC functions under ischemic stress, MSCs were cultured in serum-free medium under hypoxia (2% O_2_) to mimic the ischemic environment and treated with or without Plg. Our data showed that the apoptosis of untreated MSCs was slightly increased under hypoxia (Figure 1C). Likewise, hypoxia-induced cellular apoptosis was alleviated by the treatment with Plg (Figure 1D and Supplemental Figure 2). Together, these findings suggest that Plg promotes MSC proliferation, survival, and migration under normoxia and hypoxia in vitro.

### Plg is critical for MSC-mediated tissue repair after ischemia.

**Figure 2:**
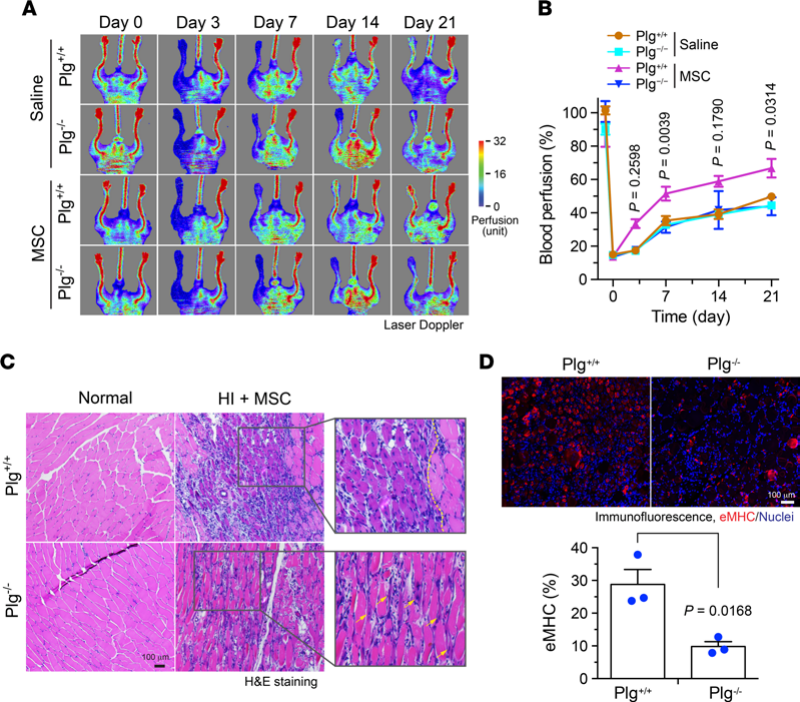
Plg is critical for MSC-mediated tissue repair after HI. HI was induced in *Plg^+/+^* and *Plg^−/−^* mice by ligation of the femoral artery, followed by MSC transplantation or saline injection in the ischemic limbs. (**A** and **B**) Mice were subjected to analysis by laser Doppler scanning. (**A**) Representative images are shown. (**B**) Blood perfusion rates in ischemic limbs were quantified and expressed as the percentage of normal limbs (*n* = 6–8, mean ± SEM). Statistical analysis by 2-way ANOVA (comparing MSC-treated *Plg^+/+^* and *Plg^−/−^* mice). (**C**) Gastrocnemius muscle in normal or ischemic limbs was collected day 7 after the surgery and subjected to H&E staining. Arrows or left area of dashed line indicate newly regenerated muscles. Original magnification, ×100. (**D**) Gastrocnemius muscle in ischemic limbs was collected at day 7 after HI surgery. Tissue sections were probed with eMHC antibody. Upper, representative images. Bottom, quantitative analysis (*n* = 3, mean ± SEM). Statistical analysis by unpaired Student’s *t* test.

To investigate the role of Plg in MSC-mediated tissue repair after ischemia in vivo, *Plg^+/+^* and *Plg^−/−^* mice were subjected to HI surgery, followed by local injection with PBS or MSCs. Our data show that MSC transplantation substantially improved blood perfusion in the ischemic limbs of *Plg^+/+^* mice, but not in *Plg^−/−^* mice (Figure 2, A and B), suggesting that Plg is required for MSC-mediated blood reperfusion in the ischemic tissues. Furthermore, gastrocnemius muscles in the ischemic tissues were pathologically examined by H&E staining and immunostaining with an antibody against embryonic myosin heavy chain (eMHC), a differentiation marker of skeletal muscle used for labeling newly regenerated muscle in ischemic tissue (23). There was no intrinsic difference in muscle development in Plg^+/+^ and Plg^−/−^ mice; however, in MSC-treated groups, skeletal muscle regeneration was robustly reduced in Plg^−/−^ mice, compared with Plg^+/+^ mice, as indicated by newly generated muscles (cells with central nuclei) (24), shown in the left area of the dashed line and by the white arrows in Figure 2C. Moreover, muscle generation, indicated as eMHC-positive muscle cells, was markedly decreased by 65% in *Plg^−/−^* mice (Figure 2D). These results together suggest that Plg is critical for MSC-mediated tissue regeneration after HI.

### Plg is required for MSC survival and persistence in ischemic tissues in vivo.

**Figure 3:**
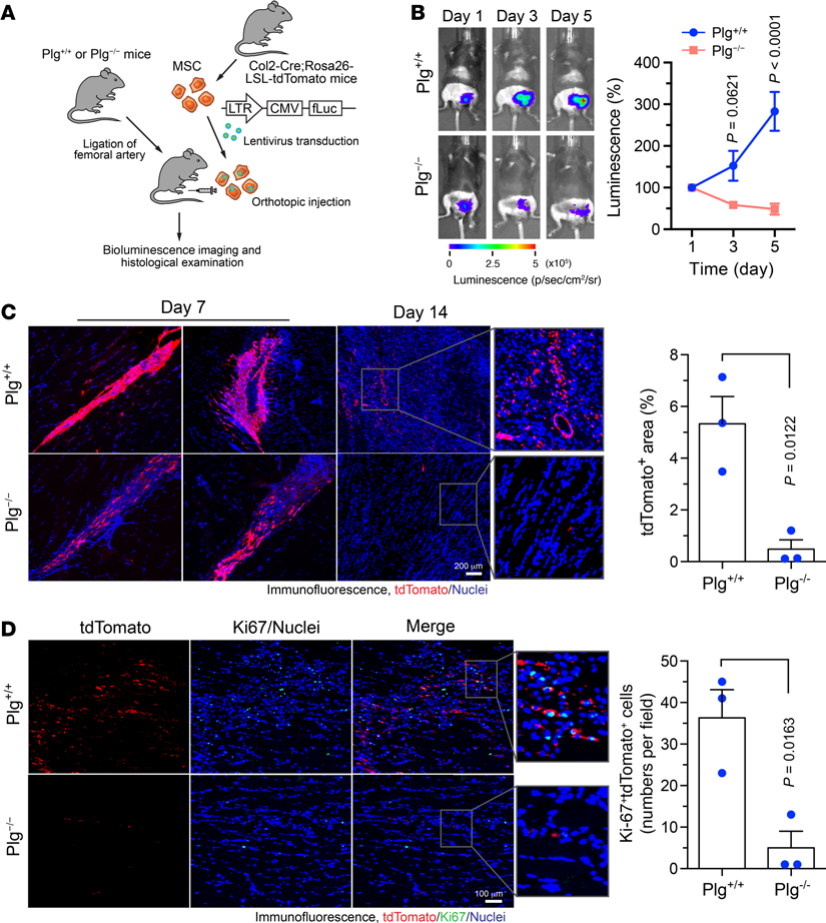
Plg is required for MSC survival, persistence, and proliferation in ischemic tissues. HI was induced in *Plg^+/+^* and *Plg^−/−^* mice by ligation of the femoral artery, followed by MSC transplantation in the ischemic limbs. MSCs were harvested from *Col2-Cre* *Rosa26-LSL-tdTomato* mice and lentivirally transduced to express firefly luciferase (fLuc). (**A**) Schematic approach. (**B**) Mice were subjected to analysis by whole-body bioluminescence imaging. Left, representative images are shown. Right, quantitative analysis of integrated luminescence (*n* = 5–6, mean ± SEM). Statistical analysis by 2-way ANOVA (comparing *Plg^+/+^* and *Plg^−/−^* mice). (**C** and **D**) Gastrocnemius muscle in ischemic limbs was collected at days 7 and 14 after the surgery. Tissue sections were probed with (**C**) anti-tdTomato antibody or (**D**) anti-tdTomato and anti–Ki-67 antibodies, followed by immunofluorescence imaging. Left, representative images. Right, quantitative analysis (*n* = 3, mean ± SEM). Original magnification, ×100. Statistical analysis by unpaired Student’s *t* test.

We next tested whether Plg regulates MSC survival and persistence in ischemic tissues in vivo. Before transplantation, MSCs isolated from *Col2-Cre* *Rosa26-LSL-tdTomato* mice were lentivirally transduced to express firefly luciferase (fLuc) for cell tracking in vivo (Figure 3A). Whole-body bioluminescence imaging analysis showed that MSCs survived in the ischemic tissues and almost tripled their numbers in 5 days after transplantation into *Plg^+/+^* mice, while Plg knockout abolished MSC survival and persistence in the ischemic limbs (Figure 3B). Likewise, immunofluorescence analysis of the ischemic muscle sections revealed that these tdTomato^+^ MSCs had decreased population (with a decrease by about 80%) and shorter persistence in *Plg^−/−^* mice than *Plg^+/+^* mice (Figure 3C). Interestingly, the tdTomato^+^ MSCs in *Plg^−/−^* mice exhibited less spreading distribution pattern, implicating reduced migration ability of MSCs in *Plg^−/−^* mice. Furthermore, Plg knockout substantially reduced MSC proliferation in the ischemic tissue, as indicated by a decrease by 80% in tdTomato^+^ MSCs expressing Ki-67, a proliferative marker, in *Plg^−/−^* mice (Figure 3D). These data suggest that consistent with its in vitro role in MSC proliferation and survival (Figure 1), Plg is required for MSC survival and persistence in ischemic tissues after HI.

### MSC-mediated neovascularization is dependent on Plg in ischemic tissues.

**Figure 4:**
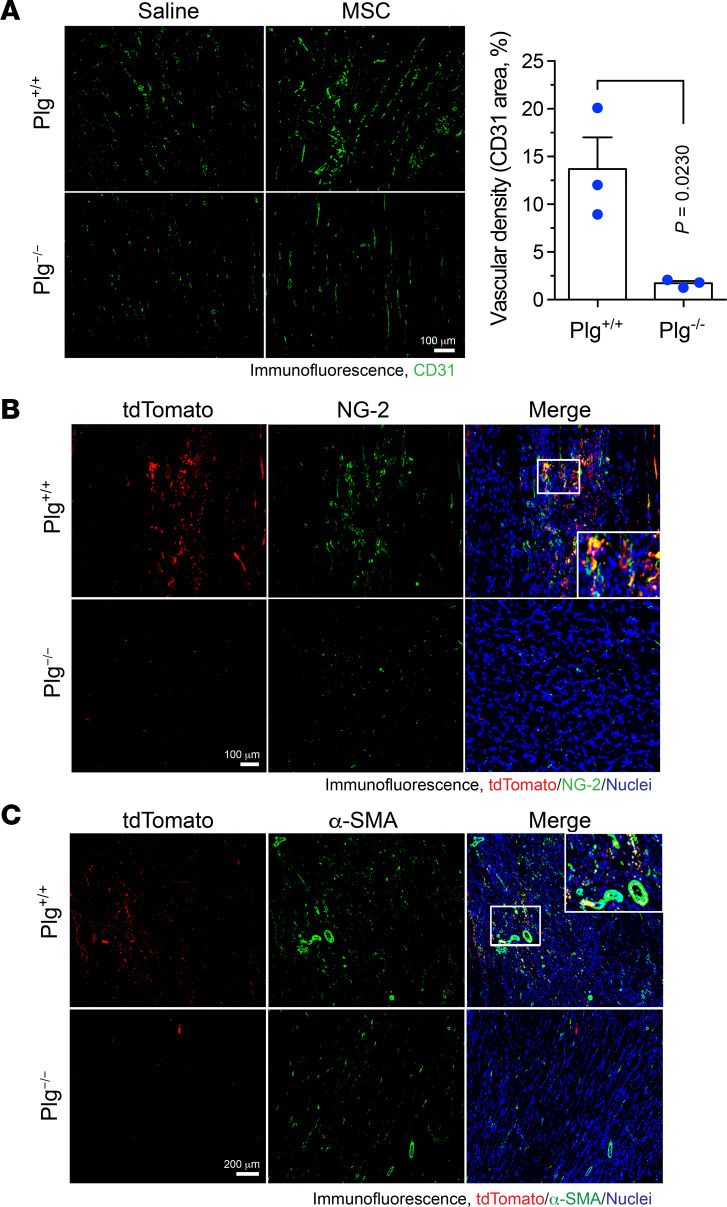
Plg is required for blood vessel formation mediated by MSC in ischemic tissues. HI was induced in *Plg^+/+^* and *Plg^−/−^* mice by ligation of the femoral artery, followed by MSC transplantation or saline injection in the ischemic limbs. MSCs were harvested from *Col2-Cre* *Rosa26-LSL-tdTomato* mice. Gastrocnemius muscle in ischemic limbs was collected at day 7 after HI surgery. Tissue sections were probed with (**A**) anti-CD31 antibody, (**B**) anti-tdTomato and anti–NG-2 antibodies, or (**C**) anti-tdTomato and anti–α-SMA antibodies, followed by immunofluorescence imaging. (**A**) Left, representative images. Right, quantitative analysis of CD31^+^ area for mice treated with MSCs (*n* = 3, mean ± SEM). Statistical analysis by unpaired Student’s *t* test. (**B** and **C**) Representative images of ischemic tissues from MSC-treated mice are shown. Original magnification, ×100.

MSCs are known to induce neovascularization and improve the local microenvironment to promote postischemic tissue repair (8). We investigated the role of Plg in MSC-mediated neovascularization after HI. Immunofluorescence studies showed that MSC transplantation enhanced neovascularization in the ischemic tissues of *Plg^+/+^* mice and that Plg deficiency almost completely abolished MSC-stimulated neovascularization (Figure 4A). Moreover, blood vessel maturation requires neural/glial antigen 2–positive (NG-2^+^) pericyte coverage, and recruitment and interaction of α–smooth muscle actin–positive (α-SMA^+^) vascular smooth muscle cells (VSMCs) further improve the vessel integrity. MSCs are known to regulate neovascularization by differentiating into pericytes and VSMCs (25). Our data show that Plg knockout almost completely abrogated the population of tdTomato^+^ MSC-derived NG-2^+^ pericytes (Figure 4B) and robustly inhibited the numbers of α-SMA^+^ cells in MSCs (Figure 4C), collectively suggesting a critical role for Plg in vascular formation and maturation (Figure 4, A–C).

### Plg activates Cyr61 by cleavage to promote MSC survival and migration.

**Figure 5:**
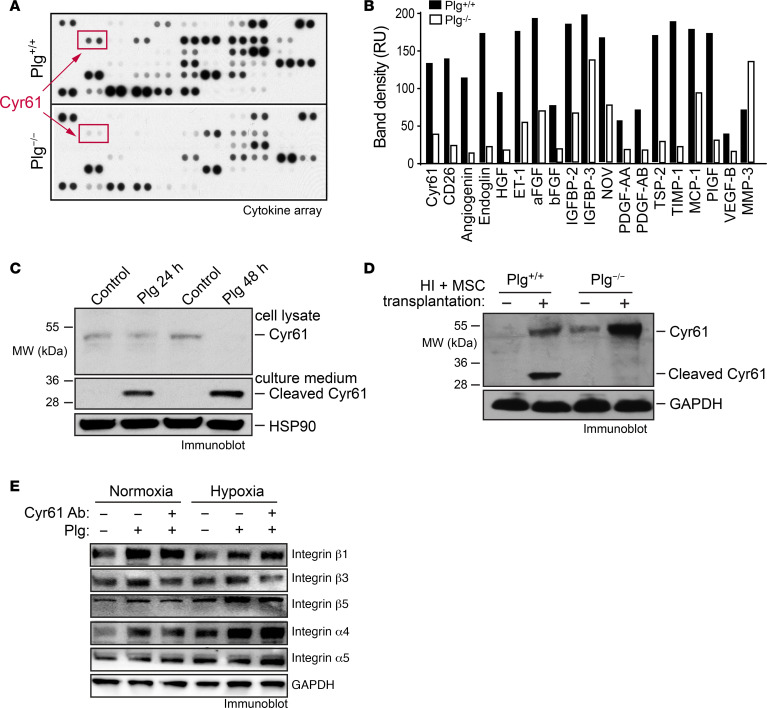
Plg cleaves and liberates Cyr61 from MSCs. HI was induced in *Plg^+/+^* and *Plg^−/−^* mice by ligation of the femoral artery, followed by MSC transplantation into the left limbs. Gastrocnemius muscle from the ischemic left limbs’ or control right limbs’ tissues was collected at day 7 after HI surgery. (**A** and **B**) Tissue lysates of the ischemic tissues were subjected to multiplex cytokine analysis. (**A**) Blotting image. (**B**) Quantified dot intensity of the most significantly changed cytokines. (**C**) MSCs were treated with or without 20 μg/mL Plg for 24 and 48 hours. Culture medium and cell lysates were immunoblotted. (**D**) Tissue lysates of ischemic tissues and control healthy tissues were immunoblotted. (**E**) MSCs were treated with Plg (20 μg/mL) in the absence or presence of anti-Cyr61 antibody (20 μg/mL) for 48 hours. Cell lysates were immunoblotted.

To explore the mechanism(s) by which Plg induces MSC-mediated neovascularization and tissue repair after ischemia, ischemic tissue lysates from *Plg^+/+^* and *Plg^−/−^* mice were subjected to multiplex angiogenic factor analysis. Our data showed that Plg altered expression of multiple angiogenic factors and growth factors (Figure 5, A and B). Among the top upregulated factors by Plg, Cyr61 is an ECM-associated growth factor, which has been previously identified as a critical regulator for MSC-regulated neovascularization in vivo (22). To bind to its receptors, Cyr61 has to be liberated from ECM by proteolysis (26, 27). Plasmin has been shown to be able to release Cyr61 from ECM by cleavage (28). Therefore, we hypothesized a potential role of Cyr61 in the Plg-dependent, MSC-mediated neovascularization and tissue repair after HI. Immunoblot analysis showed that Plg robustly induced Cyr61 cleavage in MSCs (Figure 5C), confirming the direct effects of Plg on Cyr61 cleavage and activation. Moreover, we analyzed the expression of Cyr61 in healthy and ischemic muscles in *Plg^+/+^* and *Plg^−/−^* mice after MSC transplantation. Consistent with our in vitro results, our data showed a robust Cyr61 cleavage in ischemic limb tissues of *Plg^+/+^* mice but not *Plg^−/−^* mice, which was selectively induced after MSC transplantation (Figure 5D), indicating a critical role of Plg for Cyr61 cleavage in transplanted MSCs in the ischemic tissues.

Cyr61 is known to be a ligand to integrins, including integrin αvβ1, αvβ3, and αvβ5 (29–31). Cyr61 can also trigger a Cyr61-integrin autocrine loop, in which Cyr61 stimulates integrin expression and contributes to tumor growth and angiogenesis (32). To investigate which integrins were involved in Plg/Cyr61-regulated cell function, MSCs were treated with Plg or Plg plus Cyr61-neutralizing antibody under normoxia and hypoxia, and integrin expression was analyzed. Immunoblot results showed that Plg increased expression of α4, β1, β3, and β5 integrins in MSCs under both normoxia and hypoxia conditions (Figure 5E). However, Cyr61-neutralizing antibody abrogated Plg-induced integrin β3 and β5 expression under normoxia and hypoxia, indicating that Plg/Cyr61 may regulate cell proliferation and migration through integrin β3 and β5.

### Plg-regulated Cyr61 activation is required for cell proliferation and migration in MSCs.

**Figure 6:**
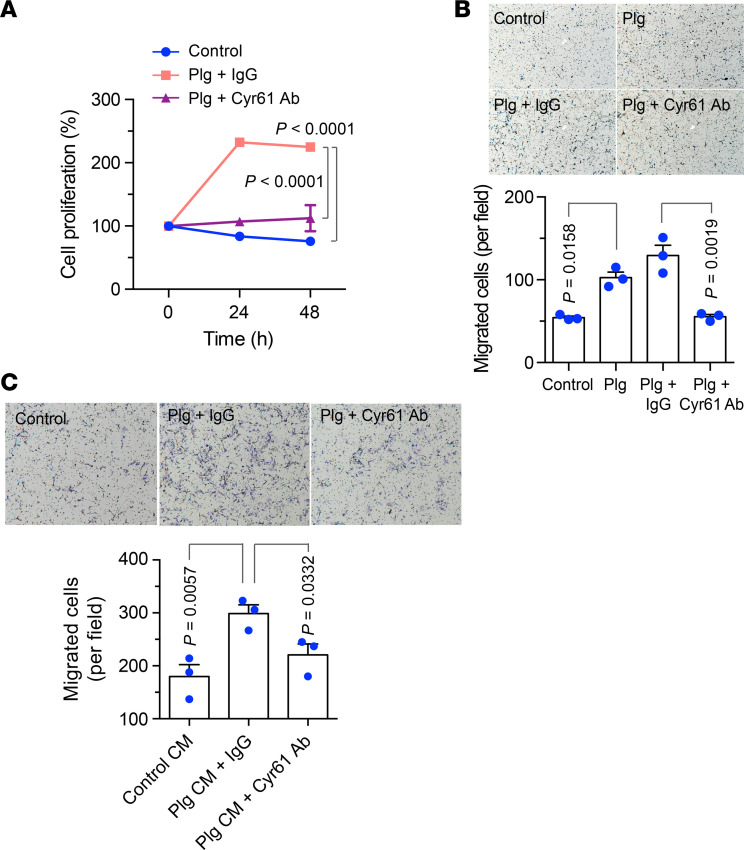
Plg activates Cyr61 by cleavage to stimulate cell proliferation and migration. (**A**) MSCs were cultured with serum-free medium and treated with or without 20 μg/mL Plg plus IgG or Plg plus anti-Cyr61 antibody under hypoxia (2% O_2_). Cell proliferation was determined by MTS-based assay (*n* = 3, mean ± SEM). Statistical analysis by 1-way ANOVA. (**B**) MSCs were treated with PBS, Plg, Plg plus IgG, or Plg plus anti-Cyr61 antibody. Plg-depleted FBS was used as a chemoattractant. Upper, representative images. Bottom, quantitative analysis (*n* = 3, mean ± SEM). Original magnification, ×100. Statistical analysis by 2-way ANOVA. (**C**) ECs were treated with control CM, Plg CM plus IgG, or Plg CM plus anti-Cyr61 antibody. Plg-depleted FBS was used as the attractant to induce the migration through matrix. Upper, representative images. Bottom, quantitative analysis (*n* = 3, mean ± SEM). Original magnification, ×100. Statistical analysis by 1-way ANOVA. Experiments were repeated 3 times and representative results are shown.

We tested the role of Cyr61 in the Plg-mediated viability and motility in MSCs. Our data showed that antibody-based Cyr61 neutralization almost completely blocked Plg-stimulated MSC proliferation (Figure 6A). Moreover, Cyr61 neutralization also abolished Plg-induced MSC migration (Figure 6B). Together, these results suggest that Cyr61 is critical for Plg-stimulated MSC proliferation and migration. Considering that Cyr61 secreted by MSCs promotes EC migration and vascular morphogenesis (22), we also investigated the role of the Plg-mediated cleavage of Cyr61 in the MSC-mediated neovascularization. Our data showed that the conditioned medium (CM) harvested from Plg-treated MSCs significantly enhanced EC migration, which was abolished by antibody-based Cyr61 neutralization (Figure 6C), suggesting that Plg cleaves and liberates Cyr61 secreted by MSCs to induce EC migration.

### Plg-induced Cyr61 activation is critical for EC migration in vivo and MSC-induced blood reperfusion after HI.

**Figure 7:**
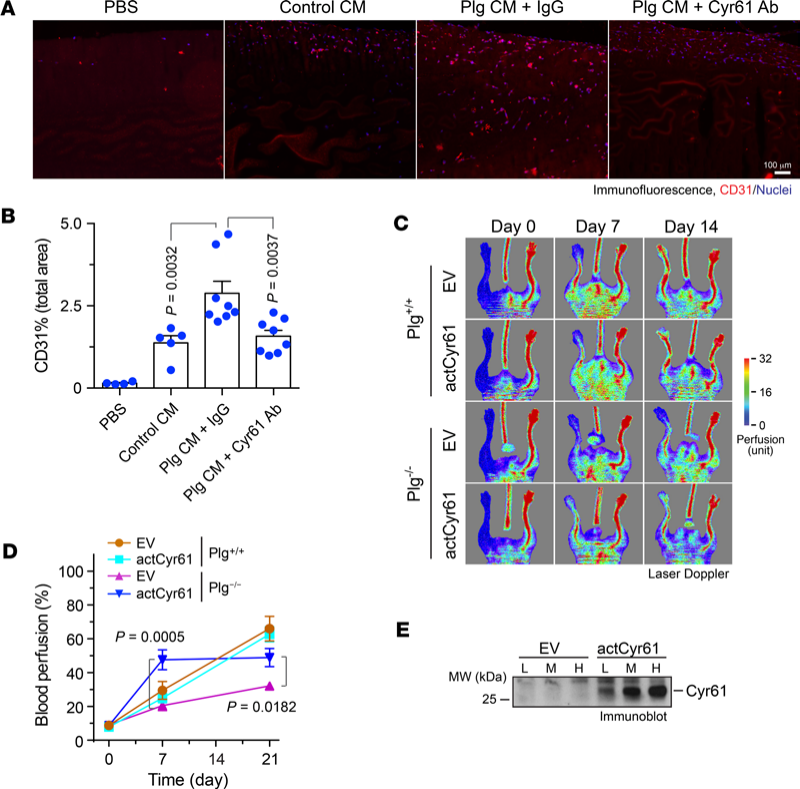
Plg-activated Cyr61 is critical for angiogenesis in vivo and blood reperfusion after HI. (**A** and **B**) Matrigel supplemented with PBS, control CM (CM derived from MSCs), or Plg CM (CM derived from MSCs treated with Plg) plus IgG, or Plg CM plus anti-Cyr61 antibody, was implanted subcutaneously in Plg^−/−^ mice. Sections of Matrigel plugs were probed with anti-CD31 antibody, followed by immunofluorescence analysis. (**A**) Representative images. (**B**) Quantitative analysis (*n* = 4–8, mean ± SEM). Statistical analysis by 1-way ANOVA. (**C**–**E**) MSCs were lentivirally transduced with Lenti-actCyr61 or empty vector (EV), followed by transplantation into ischemic limbs of *Plg^+/+^* and *Plg^−/−^* mice immediately after HI surgery. Blood perfusion was analyzed by laser Doppler scanning. (**C**) Representative images are shown. (**D**) Blood perfusion rates in ischemic limbs were quantified and expressed as the percentage of normal limbs (*n* = 3–6, mean ± SEM). Statistical analysis by 2-way ANOVA. (**E**) Cell lysates from transduced MSCs were immunoblotted. L (low), M (medium), and H (high) indicate different doses of virus used in lentiviral transduction.

To test whether Plg/Cyr61 regulates EC migration in vivo, we used a Matrigel implant model to examine EC migration and neovascularization in *Plg^−/−^* mice that minimized the background effects of Plg. Our studies showed that the CM harvested from Plg-treated MSCs robustly promoted EC migration into the implanted Matrigel (Figure 7, A and B). Notably, anti-Cyr61 antibody abolished Plg treatment–increased EC migration, indicating that Cyr61 is required for the Plg-dependent, MSC-induced EC migration.

We finally tested the role of activated Cyr61 in Plg-mediated function recovery after HI. To this end, MSCs were lentivirally transduced to express a truncated, active form of Cyr61 (actCyr61), followed by transplantation to ischemic limbs of *Plg^+/+^* and *Plg^−/−^* mice immediately after HI surgery. Laser Doppler scanning showed that expression of actCyr61 substantially increased recovery of blood perfusion in *Plg^−/−^* mice (2.4- and 1.6-fold in 1 and 3 weeks postsurgery, respectively) but did not affect blood perfusion in *Plg^+/+^* mice (Figure 7, C and D). In addition, immunoblotting results showed robust expression of actCyr61 in a dose-dependent manner (Figure 7E). Together, these data suggest that Plg-activated Cyr61 is critical for MSC-mediated angiogenesis and tissue repair after ischemia.

## Discussion

MSC therapy has emerged as a promising approach for treating ischemic diseases. Several clinical trials have shown that administration of either autologous or allogeneic MSCs to patients with acute MI reduces perfusion defect and myocardial scar and improves left ventricular ejection fraction and left ventricular remodeling (8–10, 33–36). However, a more recent clinical trial with a larger number of patients with advanced heart failure shows that intramyocardial injections of MSCs do not increase clinical benefits and patient survival, largely due to poor retention and survival of MSCs in the myocardium (13). Likewise, clinical trials show that MSC therapies effectively improve wound healing and perfusion in patients with critical limb ischemia but do not have significant effects on amputation rates (12). The transplanted MSCs survive briefly in the host and are hardly identified after a few days (37). Genetic engineering and cell pretreatment have been considered as strategies to prolong the life span of transplanted MSCs in the ischemic tissues (38–40). In this study, we reveal that Plg is critical for the survival and persistence of MSCs in ischemic limbs, and Plg plays an essential role in MSC-mediated neovascularization, therefore representing a vital therapeutic target for improving MSC therapy efficacy in ischemic diseases.

Plg is the main enzyme responsible for fibrinolysis. As a thrombolytic agent, tPA has been used as the first line of treatment of acute MI for almost 2 decades. In addition to its canonical function, Plg is critical for cardiac repair after MI, wound healing, and liver injury (17, 41, 42). Our previous work shows that Plg regulates cardiac repair after MI through promoting hematopoietic stem cell homing to the infarcted heart (17). Here, we identify an additional noncanonical function of Plg, namely, that Plg enhances MSC proliferation and migration under normoxia and improves MSC survival under hypoxia, thereby promoting MSC-mediated neovascularization and tissue repair in the ischemic tissues.

The maintenance of highly proliferative and prosurvival capacities for MSCs in ischemic tissues is essential for the success of MSC-based regenerative therapy, which is spatiotemporally subjected to regulation by multiple factors (43–45). However, the precise regulatory mechanisms are still largely unknown. In the present study, we uncover that Plg promotes MSC proliferation, migration, and survival in vitro as well as in ischemic microenvironments, suggesting Plg as a critical regulatory factor of MSC-mediated tissue repair. MSCs are a heterogeneous population of multipotent cells with the potential to differentiate into a variety of cells, including ECs, VSMCs, and pericytes, that are essential to neovascularization (6), which is regulated by multiple cytokines (46, 47). We show that Plg is critical for MSC-mediated neovascularization and tissue repair after HI, likely attributed to Plg-dependent MSC survival and persistence in the ischemic tissues and/or potentially regulated MSC differentiation into these cell types.

MSCs improve postinjury neovascularization and tissue repair, due to not only their differentiation into different cell types, but also due to their ability to alter the tissue microenvironment niche via paracrine effects on tissue repair (48). The underlying cellular and molecular mechanisms remain largely unknown, which may represent key therapeutic targets for regenerative medicine. Here, we reveal a molecular mechanism for MSC-mediated tissue repair, by which Plg activates Cyr61 in MSCs, enhancing MSC proliferation and migration and leading to MSC-mediated neovascularization. Consistent with our findings, previously published studies show that MSCs express and secrete Cyr61 to promote EC migration and vascular morphogenesis (22). Moreover, Cyr61 regulates various other biological activities, such as cell adhesion and proliferation (49, 50). Cyr61 is an ECM-associated growth factor that has to be released from ECM before it acts on cells (26, 27). A previous report shows that plasmin, the active form of Plg, activates Cyr61 by cleavage (28), in agreement with our studies showing that Plg cleaves Cyr61 in MSCs and MSC-transplanted tissues. Importantly, we demonstrate a requisite role of Plg/Cyr61 in MSC-mediated tissue repair, as evidenced by (a) that when neutralizing Cyr6, Plg fails to stimulate MSC proliferation or migration or EC migration and neovascularization and (b) that overexpression of active Cyr61 in MSCs efficiently rescues impaired recovery of blood perfusion in Plg-deficient mice after ischemia. These results support that MSCs regulate neovascularization and tissue repair through both their autocrine and paracrine function mediated by Plg-mediated cleavage of Cyr61.

In summary, we elucidate a distinct mechanism underlying MSC-mediated tissue repair and neovascularization after HI. In this newly identified pathway, Plg activates Cyr61 to promote MSC proliferation, survival, and migration, leading to neovascularization and tissue repair. Thus, targeting Plg/Cyr61 may offer promising therapeutic strategies for strengthening MSC therapy in regenerative medicine.

## Methods

### Mice.

Plg-deficient (*Plg^−/−^*) mice were generated and backcrossed into the C57BL/6J background and genotyped as previously described (51). Mice were bred, housed in sterilized isolator cages, maintained on a 14-hour light/10-hour dark cycle, and provided sterilized food and water ad libitum in the Association for the Assessment and Accreditation of Laboratory Animal Care–accredited animal facility of the University of Pennsylvania. Both male and female WT (*Plg^+/+^*) and *Plg^−/−^* mice were used between 6 and 9 weeks of age.

### Isolation and transduction of MSCs.

MSCs were freshly isolated from bone marrow of C57BL/6 mice as previously described (52) and cultured in α-MEM (SH30265-01, Thermo Fisher Scientific), supplemented with 0.1% 2-mercaptoethanol, 1% l-glutamine, and 15% FBS at 37°C in a humidified air atmosphere with 5% CO_2_. Mouse primary cardiac ECs were purchased from Cell Biologicals (C57-6024) and maintained in Endothelial Cell Medium (1001, ScienCell) at 37°C in a humidified air atmosphere with 5% CO_2_. MSCs were identified by FACs using anti–Sca-1, anti-CD90, anti-CD11b, and anti-CD117 antibodies (BD Biosciences). All cells were used between passages 2 and 5.

For tracking of MSCs in vivo or visualizing of MSCs in tissue, MSCs were isolated from *Col2-Cre* *Rosa26-LSL-tdTomato* mice (tdTomato-specific expression in MSCs, provided by Qin Ling, University of Pennsylvania, Philadelphia, Pennsylvania, USA) and were transduced with Lenti-pLEF1-pTopflash to express fLuc. Transduced tdTomato^+^ MSCs were injected locally immediately after HI, and the fate of injected MSCs was monitored by whole-body bioluminescence imaging (BLI) or visualized by immunofluorescence staining using an anti-tdTomato antibody.

### Plasmid construction and virus transduction.

Full-length and truncated (N-terminal, ~28 kDa) mouse Cyr61 were amplified by PCR using the primers (for full-length cDNA: forward primer, 5′-AGATTCTAGAGCTAGCATGAGCTCCAGCACCTT-3′; reverse primer, 5′-CAGATCCTTGCGGCCGCTTAGTCCCTGAACTTGTG-3′; for truncated cDNA: forward primer, 5′-AGATTCTAGAGCTAGCATGAGCTCCAGCACCTT-3′; reverse primer, 5′-CAGATCCTTGCGGCCGCTTACTTGGAGCACTGGG-3′) from Cyr61 (NM_010516) Mouse Tagged ORF Clone (OriGene, MR221828) as previously reported (28), followed by subcloning into pCDH-CMV-MCS-EF1-copGFP lentivirus expression vector (System Biosciences, CD511B-1). To prepare the lentivirus, HEK293T cells (CRL-3126, ATCC) were cotransfected with lentiviral expression vectors and packaging vectors (System Biosciences) for 8 hours. The medium was replaced with fresh medium; the cultures were incubated for 48 hours, followed by collection of the medium supernatants containing lentivirus. MSCs were transduced with lentivirus in the presence of 8 μg/mL polybrene (MilliporeSigma).

### Induction of HI and MSC delivery.

Unilateral HI was induced by ligation of the left femoral artery. Briefly, after anesthesia, hair removal cream was applied to remove the hair of both hind limbs, and an incision of 1 cm was made from the knee to the medial thigh on the left hind limb. The femoral artery was dissected and separated from the femoral vein and nerve. The proximal and distal femoral artery was occluded by 8-0 silk sutures with double knots. The segment of the femoral artery between the proximal and distal knots was transected. Sham-operated mice received same operation except for artery ligation. Ten million MSCs in 30 μL PBS or 30 μL PBS only (as control) were cautiously injected into the left adductor muscle using a 30-gauge insulin syringe (324910, BD Biosciences).

### Laser Doppler perfusion imaging of hind limbs.

Laser Doppler Imager (Moor Instruments) was used to serially monitor the blood perfusion recovery of the ischemic hind limbs. Briefly, mice were anesthetized and then imaged using Laser Doppler Imager. The blood flux was quantified using perfusion ratio — ratio of average laser Doppler perfusion imaging index of ischemic to nonischemic (contralateral, self-control) hind limbs — by MoorLDI V6 PC software (Moor Instruments).

### In vivo BLI for MSC tracking.

In vivo BLI was performed to track the survival of engrafted MSCs. Mice were anesthetized and received a retro-orbital injection of luciferin (150 mg/kg, LUCNA, Gold Biotechnology). Using IVIS 200 Spectrum Imaging System, images were acquired at 1-minute intervals until the peak signal was observed. Fixed-area regions of interest (ROIs) were created over left hind limbs, and photons emitted from the ROIs were quantified by photons/s/cm^2^/sr using Living Image software (Caliper Life Sciences). Animals were longitudinally imaged at 0, 1, 3, and 5 days postoperation.

### Cell proliferation and survival assays.

MSCs were suspended in α-MEM supplemented with 15% FBS and seeded on 96-well plates at a density of 6 × 10^3^ cells per well under normoxia at 37°C and allowed to attach for 16 hours. For cell proliferation, CM was replaced with α-MEM supplemented with 0.3% BSA with or without addition of Plg (human Glu-Plg, HPG2070, Enzyme Research Laboratories), and cells were incubated under normoxia at 37°C for 6 days. For cell survival, CM was replaced with α-MEM supplemented with 0.3% BSA with or without addition of Plg and IgG or anti-Cyr61 antibody (MAB4864, R&D Systems, Bio-Techne), and cells were exposed to hypoxia (2% O_2_) at 37°C for 24 or 48 hours. Cell viability was determined by MTS assay (ab197010, Abcam) according to the manufacturer’s instructions.

### Apoptosis assay.

MSCs were suspended in α-MEM supplemented with 15% FBS and seeded on 6-well plates at a density of 2 × 10^5^ cells per well and allowed to attach for 16 hours. Culture medium was then replaced with α-MEM supplemented with 0.3% BSA with the addition of Plg, and cells were incubated under hypoxia (2% or 1% O_2_) at 37°C for 24, 48, or 72 hours. Apoptosis assay was performed using the FITC Annexin V Apoptosis Detection Kit I (556547, BD Biosciences) following the manufacturer’s instructions.

### Depletion of Plg in FBS.

FBS was passed over Lysine-Sepharose 4B columns (17-0690-01, GE Healthcare) several times, and the flow-through fraction was collected (53). Depletion of Plg was confirmed by bovine plasminogen activity kit (EBP2211-1, AssayPro).

### Preparation of MSC CM.

MSCs were seeded in 6-well plates with α-MEM supplemented with 15% FBS under normoxia at 37°C. After cells were attached, medium was changed into α-MEM supplied with 0.3% BSA. MSCs were then treated with or without Plg (20 μg/mL) for 24 hours. CM was collected and centrifuged at 3000 *g* for 15 minutes to remove cellular debris, and the supernatant was stored at –80°C. Culture medium from nontreated MSCs is called control CM; culture medium from Plg-treated MSCs is called Plg CM.

### Cell migration and invasion assays.

Cell migration and invasion assays were performed by using a 24-well Transwell system (353097, Corning). MSCs were cultured in α-MEM supplemented with 0.3% BSA and seeded (3 × 10^4^ cells/well) on inserts precoated with Matrigel. Plg (20 μg/mL) with IgG or anti-Cyr61 antibody (20 μg/mL) was added into the inserts. Cell migration was induced by 1% Plg-depleted FBS in the bottom chamber. Twenty-four hours later, cells on the top of the membrane were wiped off by a cotton swab. For EC migration, ECs were seeded at a concentration of 1 × 10^5^ cells/well in control CM or Plg CM collected as described above. IgG or anti-Cyr61 antibody (20 μg/mL) was then added into Plg CM–treated groups. Cell migration was induced by α-MEM with 5% Plg-depleted FBS in the bottom chamber. Six hours later, cells on the top of the membrane were wiped off by a cotton swab. Membrane of inserts were then fixed in methanol and stained with toluidine blue (198161, MilliporeSigma) for 5 minutes. Images were taken in 3~4 fields per well under a microscope (Axio Lab A1, ZEISS), and migrated cells on the bottom of the membrane were counted.

### Matrigel plug assay.

MSC CM was concentrated from 500 μL to 50 μL by Amicon Ultra filters (UFC5003, MilliporeSigma). The concentrated CM with IgG or anti-Cyr61 antibody was mixed with Matrigel (356231, Corning) to a final volume of 500 μL on ice, and the mixture was then implanted subcutaneously in the dorsal area of *Plg^−/−^* mice. Seven days after implantation, the Matrigel plugs were collected and embedded in paraffin for immunostaining.

### Cytokine array.

Ischemic tissues were collected from *Plg^+/+^* and *Plg^−/−^* mice 1 week after surgery. Tissues were lysed with an NP-40 buffer containing protease inhibitor cocktail (11697498001, Roche). Tissue lysates were subjected to multiplex cytokine analysis using a mouse angiogenesis array kit (ARY015, R&D Systems, Bio-Techne) according to the manufacturer’s instructions.

### Immunoblot.

Cells and tissues were lysed with a NP-40 buffer containing protease inhibitor cocktail. Concentrated CM or cell or tissue lysate was resolved by 4%–15% precast SDS-PAGE gel (Bio-Rad). After transfer, PVDF membranes were blotted with anti-GAPDH (1:5000, catalog 5174, Cell Signaling Technology), anti-HSP90 (1:1000, catalog 4874, Cell Signaling Technology), anti-Cyr61 (1:1,000, catalog MAB4864, R&D Systems, Bio-Techne), anti-CXCR4 (1:500, catalog 1670, Abcam), or anti-integrins (1:1000, catalog 4749, Cell Signaling Technology) antibodies. Proteins were detected with secondary antibodies specific for either rabbit (catalog 170-6515, Bio-Rad) or mouse (catalog 170-6516, Bio-Rad) IgG, followed by ECL development (RPN2232, GE Healthcare).

### Immunofluorescence and histology.

Paraffin sections were deparaffinized and rehydrated and subjected to antigen retrieval in Antigen Retrieve Solution (HK086-9K, Biogenex) at 95°C for 20 minutes. Sections were blocked with 5% horse serum for 1 hour at room temperature. Mouse tissues were incubated with anti-CD31 (1:100, catalog DIA-310, dianova), anti-tdTomato (1:100, catalog MBS448092, MyBioSource), anti–Ki-67 (1:100, catalog AB9260, MilliporeSigma), anti–NG-2 (1:100, catalog AB5320, MilliporeSigma), anti–α-SMA (1:100, catalog A5228, MilliporeSigma), and anti-eMHC (catalog F1.652, DSHB) antibodies overnight at 4°C. For Matrigel plug assay, sections were incubated with anti-CD31 antibody overnight at 4°C. Sections were stained with Alexa Fluor 488–conjugated (catalog A-21202, catalog A-11006, catalog A-11034, Thermo Fisher Scientific), 568–conjugated (catalog 11079, catalog A-11004, Thermo Fisher Scientific), and 647–conjugated (catalog 405416, BioLegend) appropriate secondary IgGs (1:500) for 1 hour at room temperature. Images were acquired with an Axio Imager fluorescence microscope (ZEISS). Images were quantified by ImageJ software (NIH). For histological study, sections were stained with H&E and imaged with an Axio Lab microscope (ZEISS).

### Statistics.

Two-tailed Student’s *t* test and 1-way or 2-way ANOVA were used for statistical analysis between groups, and *P* values less than 0.05 were considered statistically significant.

### Study approval.

All animal experiments were performed in accordance with protocols approved by the Institutional Animal Care and Use Committee at University of Pennsylvania.

## Author contributions

HD and ZH performed and analyzed experiments and produced figures. ML performed image acquisition, quantification, and immunoblotting. YW constructed plasmids. FY performed Western blotting on integrins. RAM contributed to cell proliferation analysis. HJK and EY contributed to blood perfusion data analysis in HI experiments. HH contributed to cytokine array analysis. LQ and YF provided suggestions for experimental design. YG conceived the concept, designed the experiments, supervised the project, and wrote the manuscript. All authors commented on the manuscript.

## Supplementary Material

Supplemental data

## Version 1 08/06/2020

Electronic publication

## Version 2 08/11/2020

Affiliations update
