# Supplementary material for: Plasminogen regulates mesenchymal stem cell–mediated tissue repair after ischemia through Cyr61 activation
Source: JCI Insight. 2020 Aug 6;5(15):e131376. doi: 10.1172/jci.insight.131376 (PMC7455064; doi:10.1172/jci.insight.131376)
Supplement: Supplemental data [file jciinsight-5-131376-s264.pdf]

A

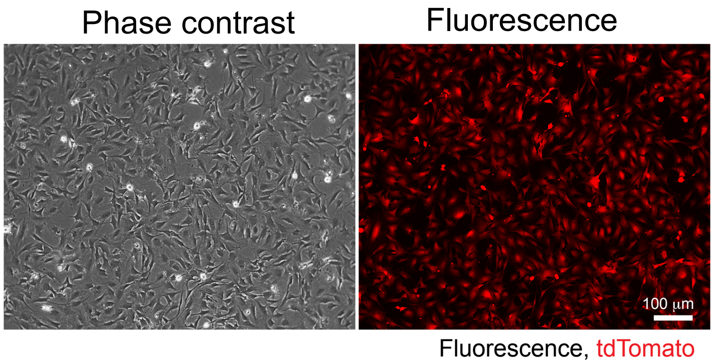

B

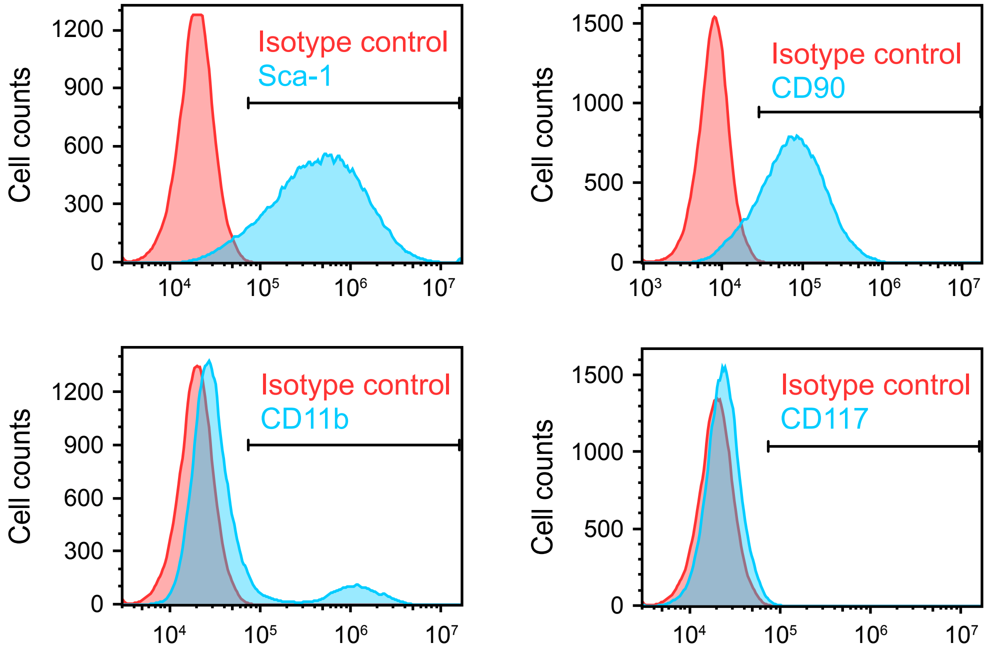

Supplementary Figure 1. Characterization of isolated MSCs.
MSCs were isolated from bone marrow of *Col2-Cre*;*Rosa26-LSL-tdTomato* mice. (**A**) Cells were imaged by light or fluorescence microscope. Representative images are shown. (**B**) Cells were probed with anti-Sca-1, anti-CD90, anti-CD11b, or anti-CD117 antibody, and analyzed by flow cytometry. Representative sortings are shown.

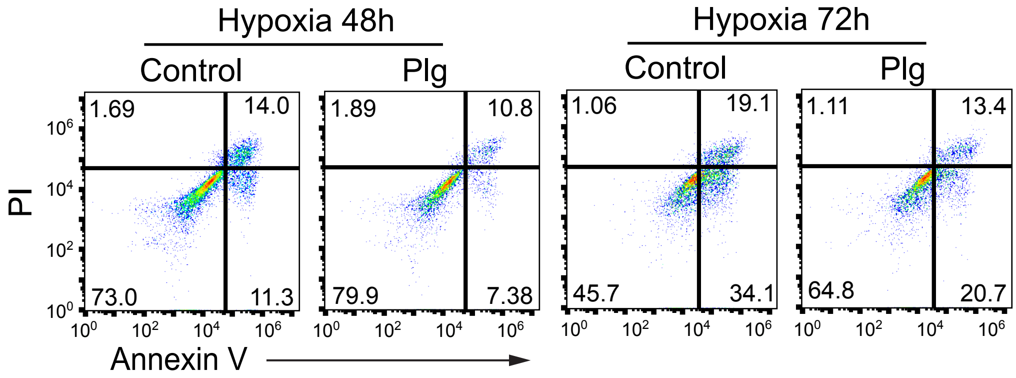

Supplementary Figure 2. Plg inhibits hypoxia-induced apoptosis in MSCs.
Mouse MSCs were treated with 20 μg/ml Plg or control medium under hypoxia (1% O_2) for 48 or 72 hours. Cells were stained with propidium iodide (PI) and annexin V, followed by flow cytometry analysis.
